# Perceived psychosocial stressors and coping resources in chronic low back pain patients as classified by the avoidance-endurance model

**DOI:** 10.3389/fresc.2022.996945

**Published:** 2022-10-31

**Authors:** Elisabeth Fehrmann, Linda Fischer-Grote, Thomas Kienbacher, Kerstin Tuechler, Patrick Mair, Gerold Ebenbichler

**Affiliations:** ^1^ Department of Psychology, Karl Landsteiner Institute of Outpatient Rehabilitation Research, Vienna, Austria; ^2^ Rehab Zentrum Liesing, Karl Landsteiner University of Health Sciences, Krems, Austria; ^3^ Department of Psychology, Harvard University, Cambridge, MA, United States; ^4^ Department of Physical Medicine, Rehabilitation and Occupational Medicine, Vienna Medical University, Vienna, Austria

**Keywords:** chronic low back pain, avoidance-endurance, psychosocial stressors, coping resources, subgroups

## Abstract

**Objectives:**

The Avoidance-Endurance Model distinguishes between subgroups of low back pain (LBP) patients with three maladaptive styles of coping with pain: fear-avoidance (FAR), distress-endurance (DER), eustress-endurance (EER), and one adaptive coping style (AR). This study aimed to compare the quantity of patients' perceived psychosocial stressors and coping resources across these subgroups.

**Materials and methods:**

This cross-sectional study was conducted at an outpatient rehabilitation center for patients with chronic musculoskeletal pain. One hundred and thirty-seven patients (69 women/68 men) with chronic LBP were assessed using the following: a demographic checklist, the visual analogue scale, Avoidance-Endurance Questionnaire, Roland-Morris Disability Questionnaire, Pain Disability Index, and 36-Item Short Form. Subsequently, patients participated in semi-structured interviews led by clinical psychologists, which were intended to identify their perception of stressors and coping resources. The quantity of psychosocial stressors and coping resources were analyzed using deductive and inductive content analyses and then compared between subgroups using chi-square-tests.

**Results:**

FARs experienced significantly higher levels of “mental suffering” (*p* = <0.001) and “other workplace problems” compared to ARs and EERs (*p* = <0.001). DERs reported significantly higher levels of “mental suffering” (*p* = <0.001), “job stress” (*p* = 0.022), and “familial losses” (*p* = 0.029) compared to ARs, whereas the AR group demonstrated significantly more “coping resources” (*p* = 0.001) compared to FARs.

**Conclusion:**

AEM-subgroups differed in the quantity of perceived psychosocial stressors and coping resources with AR, who demonstrated a lower risk for pain chronicity and reported the highest quantity of resources. The variability across subgroups may imply differences in patientś needs regarding therapeutic interventions and suggests that a resource-centered approach to cope with stress and pain may be beneficial.

## Introduction

The Avoidance-Endurance model (AEM) distinguishes between four different pain coping patterns in persons afflicted with low back pain (LBP): patients with fear-avoidance (FAR), eustress-endurance (EER), distress-endurance (DER), and adaptive pain response patterns (AR) (1). The three maladaptive pain response or pain coping patterns (FAR, EER, DER) constitute a risk factor for pain chronification. Differences in the pain response are likely driven by differences in coping with pain-related fear, pain-related cognition, and affective responses (2). Patients with FAR tend to employ catastrophizing thoughts, which correspond with an increased fear of pain and the avoidance of specific movements or activities, while patients with EER or DER tend to suppress thoughts of fear and pain or distract themselves, which allows them to maintain activities despite experiencing pain (1). Patients with EER are typically in a more positive mood, whereas those with DER demonstrate a more negative mood in response to pain (1, 2). By contrast, patients who demonstrate an adaptive coping style regarding pain are able to observe bodily signals and deal with them in a flexible way, neither avoiding nor persisting too much in their activities despite pain. Patients with such adaptive coping techniques are less at risk for pain chronicity.

Next to the aforementioned three maladaptive pain response patterns, acute or chronic stress is an important risk factor for pain chronicity. Lazarus and Folkman defined stress in the cognitive-transactional model: first, a person subjectively evaluates whether a situation is dangerous, positive, or neutral (mostly on an unconscious level); second, the person assesses his or her internal (e.g., abilities and skills) or external resources (e.g., social support, etc.) to cope with the situation (3). Only if a situation is assessed as dangerous and if coping resources are considered insufficient does the person feel stressed. Stress affects the person as a whole, causing physical, mental, and emotional strain or tension (3). According to the World Health Organization, stress is the “Health Epidemic of the 21st Century” and it is responsible for devastating health problems and exorbitant costs to society (4). Daily hassles as well as hyperstress are known to modulate the intensity of pain perceived by an individual and facilitate pain persistence (5). Increased stress loads are associated with enhanced bodily tension and trunk stiffness, with possible long-term consequences like degenerative changes in the musculoskeletal system of the trunk (6, 7). Stress may also facilitate both the intensity of pain and processes within the central nervous system involved in the chronification process of pain (7, 8). Consequently, perceived stressors considered in clinical practice guidelines for back pain and their features have been recognized to be of utmost importance to pain maintenance. These stressors have been summarized in a flag system, where “yellow flags” refer to mental strain and pain related-stressors (e.g., catastrophizing thoughts and pain-related cognitions), “orange flags” include comorbidities, (e.g., anxiety or depressive disorders), and “blue flags” subsume work- and family-related/social stressors (9). Mental suffering that is frequently associated with mental illnesses and psychological comorbidities (10–13) constitutes a particularly high risk factor for pain chronification (9) and is often linked to worse therapy outcomes (14). A recent review by Rusu and co-authors found that pain responses like catastrophizing and thought suppression also mediate the relationship between depression and pain (15). Furthermore, work-related stressors (16–21) as well as family-related and social stressors were found to have an impact on both pain perception and maintenance (22–25). Thus, coping with stress is considered as highly relevant in the context of pain, and the presence and awareness of internal and external resources help patients to cope with stress as well as pain (3). Depressive patients with and without pain show different kind of biases, as patients with pain show a recall bias for illness- and health-related stimuli (15, 26). Although a general recall bias amongst pain patients towards pain-related stimuli is described in the literature (15, 27, 28), this finding may not apply to individual AEM-groups. Indeed, it has been identified that FARs tend toward avoiding pain-related stimuli (29).

Since strategies to cope with both stress and pain (such as AEM patterns) are important contributors to the maintenance of LBP, it is surprising that little research has investigated whether there are associations between and common features across these strategies. Research to date has found that FAR is associated with work absences and disabilities at work (30, 31), whereas pain persistence behaviour, a term comparable to DER (2, 32, 33), is linked to over-activity and work-related stress (34), and to anxious-ambivalent or anxious-avoidant attachment styles in relationships (35). The latter mentioned study also suggests overactive patients are likely to be more ambitious at work, which results in higher stress levels and in turn higher long-term pain levels and consequent pain-avoidance (35). In another study that measured stress levels through the cortisol awakening reaction, elevated cortisol levels associated with endurance behaviour and lower cortisol levels (and hypocortisolism) associated with fear-avoidance behaviour, possibly connected with long-term stress, were found (36). A study with 851 LBP patients found more affective distress (which is comparable to mental suffering) in the groups of FAR and DER compared to AR and EER (37).

In order to further understand the role of perceived psychosocial stressors and their relevance to groups of different LBP response patterns, this study sought to compare the quantity of patients' psychosocial stressors and coping resources across these subgroups. We hypothesized that patients with maladaptive pain response patterns (especially FAR and DER) would report higher levels of mental suffering and perceived work- and family-related/social stressors compared to ARs and EERs and less coping resources compared to ARs.

This study builds upon a study published in 2017 (38) by providing a greater depth of psychological data analysis.

## Materials and methods

Between January 2012 and July 2015 all patients who sought treatment for LBP and were referred to the outpatient physical medicine and rehabilitation center were asked to participate in a study. Of these, 216 patients agreed to participate in this cross sectional study. The inclusion criteria were: pain in the lower back with and without radiation lasting for more than three months (39) at a minimum average pain level of 30 on a visual analogue scale (VAS, 0–100) (40) and a minimum age of 18 years. Exclusion criteria were: moderate pain levels (>30 on a VAS) in areas other than the lower back; peripheral neurological deficits; spinal fractures, infection, or cancer; recent surgeries involving the back region; previous experience with trunk muscle strength testing, and a body mass index (BMI) exceeding 35 kg/m^2^. After screening for inclusion and exclusion criteria, a total of 178 CLBP patients were eligible for inclusion in the study. All patients received oral and written information about the study and signed a consent form. The study protocol was approved by the ethics committee of the city of Vienna (Thomas-Klestil-Platz 8/2, TownTown, A-1030 Vienna (EK_11_181_VK_NZ).

### Measures

Patients with CLBP were assessed once using the following questionnaires and interviews.

#### Sociodemographic, pain history and disability measures

The patients’ gender, age, education level, marital status, and pain history were all assessed with a general sociodemographic and medical history checklist. Current pain intensity was rated on visual analogue scales (VAS) ranging from 0 (no pain) to 100 (highest pain) (40). The VAS has been shown to be a valid and reliable measurement (41).

The German version of the Roland-Morris disability questionnaire (RMDQ) measures LBP-related functional disability (42). The RMDQ assesses 24 items that patients either agree (1) or disagree (0) with. The sum score ranges from 0 to 24, with higher scores indicating higher disability levels. The RMDQ has proven to be both valid and reliable (42, 43).

The Pain Disability Index (PDI) assesses experienced pain-related disability in seven life domains measured on 11-point rating scales (0 = no disability, 10 = total disability) (44). The sum score ranges between 0 and 70, with higher scores indicating higher disability. The PDI has been shown to be valid and reliable (44, 45).

#### Avoidance- and endurance pain response patterns and mental health

Avoidance-endurance behaviour was measured with the Avoidance-Endurance Questionnaire (AEQ) (1), which comprises of 49 items and nine subscales. Patients rated the items on a seven-point rating scale (0 = never, 6 = always). All of the AEQ subscales have been proven to have high levels of validity and internal consistency (1). For the AEM-subgroup classification, the thought suppression scale (TSS), the behavioural endurance scale (BES), and the Mental Health Inventory (MHI) of the 36-Item Short Form Health Survey (46, 47) were taken. The behavioural endurance scale (BES) is a sum scale calculated by the subscales of the humour/distraction scale (HDS) and the pain persistence scale (PPS) (1). The MHI measures depression and anxiety, and demonstrates excellent validity and reliability as well as good to excellent correlations with the Beck Depression Inventory (48).

#### Half-standardized, semi-structured interviews

Half-standardized, semi-structured interviews (49) led by clinical psychologists were intended to screen for and rate the quantity of the subjectively perceived individual psychosocial stressors and coping resources. The interviews followed the recommendations of previously published evidence-based guidelines, and recommendations within the literature (49–51). The interviewers were unaware of the AEM-subgroup classification. Each interview lasted approximately one hour. See Table 1 for the interview guideline. Written theme-centered notes were made during the interviews. Prior to the start of the study the feasibility and applicability of the interview procedures were tested in five interviews that were not included in the analysis.

**Table 1:** Topics of half-standardized interviews.

	Topic	Questions
1.	Pain	Actual pain intensity? Today's state of health?
2.	Explanatory model	What do you think are possible explanations for the onset of pain? Which behaviour does lead to an increase or decrease of pain?
3.	Mental health and/or mental suffering	Is there any connection between pain and your feelings? How do you feel most of the time? Do you know feelings of depressed mood or anxiety? How often do such feelings occur? Is there anything you feel stressed by?
4.	Well-being and stressors at work	How do you feel in your job? Are there any job-related stressors (like time pressure, conflicts, lack of variability and so on)?
5.	Well-being and stressors at the family and in social life	How do you feel in your family and social life? Are you satisfied within these areas of life? Are there any things you feel stressed by (conflicts, negative feelings, lack of time, illness of relatives, and so on)? Is that a burden on you?
6.	Resources/abilities to alternate pain	What resources do you have? Are there any abilities you have to alternate pain? What gives you a relief of pain? What can you do to decrease pain?

### Statistical analysis

#### Cluster analysis

According to the AEM and two recent publications (38, 52), CLBP patients were classified into the subgroups by a cluster analysis using the TSS and BES subscales of the AEQ and the MHI (1, 47). The raw scores were transformed to *z*-scores to provide standardized scores for subsequent cluster analysis. Based on an input distance matrix (Euclidean distances) a single-linkage hierarchical clustering was applied to detect and subsequently eliminate outliers. After removing the outliers, a Ward hierarchical clustering was performed to minimize the within-cluster sum-of-squares. To determine the number of clusters, statistical [NbClust package (53), cluster R-squared] as well as theoretical/substantive criteria (2, 32, 54) were taken into account. In line with the theoretical model of the AEM, LBP patients were sub-classified into: (1) FARs with low TSS, BES, and low MHI scores; (2) DERs with high values at TSS and BES and low MHI scores; (3) EERs with high scores at TSS, BES, and MHI, and, (4) ARs with low TSS, BES, and high MHI.

To validate the classification with the cluster analysis, a confirmatory analysis was conducted with AEM-subgroups based on cut-off values of BES and TSS of 3 (2), and MHI of 70 (55).

#### Qualitative analysis of content

Deductive and inductive categories were built by using the structuring content analysis developed by Mayring (56). A quantitative analysis was used to determine frequencies of categories. The main categories of the deductive analysis were “mental suffering”, “work-related stressors”, and “family-related/social stressors”. These categories were defined and selected using examples of the patients’ reports to guarantee valid ratings. In the category “mental suffering” patients’ reports were counted when they identified issues with their mental health, expressed negative thoughts, or identified feelings of depression or sustained anxiety (representative example: “I feel depressed, unmotivated. Especially now that it's winter. I do not have any strength any more”). The category “work-related stress” comprised psychological aspects of work-related stress, e.g., perceived job stress, time pressure, conflicts with colleagues, discontentment, and unemployment (representative examples: “I can hardly pause. I've earned a lot by being diligent. Time is more important to me than my back”, and, “I am afraid of downsizing.”). The “Family-related/social stressors” category comprised conflicts and quarrels in the family, discontentment, isolation, familial troubles, stress through illnesses, separations, etc. (representative example: “There are problems in the relationship, lots of screaming.”).

Inductive categories were developed from the transcripts of patients' interviews using qualitative content analysis to identify words, phrases, or conditions that were mentioned frequently or had a high impact on the patients. The following four inductive categories were developed: “job stress”, and “other workplace problems” (to subcategorize the deductive category “work-related stress”), “familial loss”, and “coping resources”. The inductive category “job stress” subsumed high work-load, time pressure, high responsibility, and lack of possibilities to relax (representative example: “when I know that there is still a lot of work to do, I can’t even relax during breaks”). In the inductive category “other workplace problems”, stressors like dissatisfaction with the workplace, absences because of illness or temporary invalid pension, unemployment, troubles finding a job and a bad working atmosphere were included (representative example: “conflicts with the staff are troubling, I am too sensitive to cope with those”). The category “familial loss” comprised experiences of loss, separation, and cases of death in the family (representative example: “my mother and my brother both died in the last two years. Due to that nothing brings joy.”). The category “coping resources” comprised internal and external resources such as coping-strategies, social support, and interests/hobbies, etc. (representative examples: “exercising regularly is good for me”, and, “I’ve got a wonderful support system”). The categories identified through deductive and inductive content analyses were counted for each interview with “true” or “untrue”.

#### Chi-square tests

To compare the frequencies in the main categories and the inductive categories between the different AEM-subgroups, Pearson's chi-square tests were calculated. The primary outcomes of this study were the differences of AEM-subgroups in the deductive categories “mental suffering”, “work-related stressors” and “family-related/social stressors”. Secondary outcomes showed the subgroup-differences in “job stress”, “other work-related stressors”, “familial loss”, and “coping resources”. *p*-values were computed by Monte Carlo simulation (57). In case of a significant chi-square test, specific cells were compared in line with the proposed hypotheses (FAR and DER have more stressors and less resources compared to EER and AR), calculating a *z*-test for the cells in questions (58). The resulting *z*-value is compared to the square root of chi-square critical value of the whole table (59). In case of a *z*-value outside the range of ± the square root of the chi-square critical value, the null hypothesis is rejected (58). Effect sizes are reported by using Cramer's *V* [interpretation based on (60): weak effect: *V* = 0.2; moderate effect: *V* = 0.5; strong effect: *V* = 0.8].

To externally validate the classification into AEM-subgroups with the cluster analysis, chi-square tests were also conducted on the AEM-subgroups based on cut-offs, another subgroup-classification method proposed by Hasenbring et al. (1).

All statistical analyses were performed in the R environment for statistical computing® (61).

## Results

### Study sample

Of the 178 CLBP patients eligible for inclusion in the study, 29 dropped out during the study, two patients had to be excluded because of missing data within the AEQ, and the single linkage clustering method proposed an exclusion of 10 extreme outliers (38). The final study sample for this cross-sectional study comprised 137 persons. The mean age of the participants was 47.20 years and 50.4% were female. The patients reported a mean pain duration of 10 years, and an average pain intensity of the last three months of 50 on a VAS scale (0–100). For further sample characteristics see Table 2.

**Table 2:** Demographics, pain, and disability of 137 chronic low back pain patients.

Characteristics	Mean (SD)	*n* (%)
Age^1^	46.89 (16.39)	
Gender		
Female		69 (50.4%)
Male		68 (49.6%)
Body mass index^2^	25.81 (4.39)	
**Marital status**		
Married		68 (50%)
Unmarried		38 (28%)
Other		31 (22%)
**Educational level^3^**		
Low		11 (8%)
Intermediate		62 (45%)
High		61 (45%)
Not specified		3 (2%)
**Employment status**		
Employed		78 (57%)
Retired		37 (27%)
Student		11 (8%)
Unemployed		6 (4%)
Self-employed		2 (2%)
Not specified		3 (2%)
**Pain characteristics**		
Pain Intensity at baseline^4^	28.50 (18.81)	
Average pain intensity last 3 months^4^	51.56 (13.23)	
Total pain duration^5^	128.90 (123.39)	
Roland Morris disability questionnaire score	6.50 (3.78)	
Pain disability index score	15.83 (10.96)	

SD, standard deviation; *n,* number; ^1^ = in years; ^2^ = in kg/m^2^; ^3 ^=^ ^low/intermediate/high educational level = 8 years/10–12 years/>12 years of schooling; ^4^ = pain scores on a visual analogue scale (0–100); ^5^ = in months.

### Results of the cluster analysis

The cluster analysis classified 24% (*n* = 33) of the patients as FARs, 34% (*n* = 47) as DERs, 17% (*n* = 23) as EERs and 25% (*n* = 34) as ARs. The means and SDs of the clustering variables are provided in Table 3.

**Table 3:** Means and SDs of the (untransformed) clustering variables by the resulting cluster*s.*

		TSS	BES	MHI
subgroup	Number of positive answers; *n* (%)	Mean (SD)	Mean (SD)	Mean (SD)
FAR	33	2.11 (1.23)	3.09 (0.58)	58.06 (9.17)
DER	47	3.62 (0.77)	3.35 (0.43)	79.15 (7.73)
EER	23	3.89 (1.16)	4.51 (0.35)	82.26 (7.12)
AR	34	0.76 (0.73)	3.12 (0.79)	86.71 (5.72)

*n,* numbers; SD, standard deviation; TSS, avoidance-endurance questionnaire thought suppression scale; BES,  avoidance-endurance questionnaire behavioral endurance acale; MHI, SF36 mental health inventory; FAR, fear-avoiders; DER,  distress-endurers; EER, eustress-endurers; AR, adaptive responders.

In the confirmatory analysis 7% (*n* = 10) of the patients were identified as FARs, 19% (*n* = 26) as DER, 59% (*n* = 81) as EER and 15% (*n* = 20) as AR.

### Results of the deductive categories: “mental suffering”, “work-related stressors” and “family-related/social stressors”

A total of 39.7% (*n* = 52) of all patients reported that they suffer mentally, 54% (*n* = 74) reported work-related stress, and 32.1% (*n* = 44) identified family-related/social stressors. For the numbers divided by AEM-subgroups see Table 4. Pearson's chi-square test revealed a moderate effect with FARs identifying significantly more mental suffering than ARs and EERs, and DERs also identifying significantly more mental suffering than ARs (Cramer's *V* = 0.411, see Table 4).

**Table 4:** Numbers of positive answers of deductive categories per AEM-subgroup and results of Pearson's Chi-Squared tests to compare the subgroups within these categories*.*

Category	Number of positive answers; *n* (%)				Pearson's Chi-Squared tests			Compared cells
	FAR	DER	EER	AR	*χ* ^2^	*p*	*V*	
Mental suffering^1^	21 (67.74)	21 (45.65)	6 (27.27)	4 (12.50)	22.174	**<0**.001*	0.411	FAR > AR and EER; DER > AR
Work-related stressors	20 (60.61)	26 (55.32)	13 (56.52)	15 (44.12)	2.008	0.559	0.121	
Family-related/social stressors	10 (30.30)	15 (31.91)	10 (43.48)	9 (26.47)	1.910	0.588	0.118	

AEM, avoidance-endurance model; *n,* number; AR,  adaptive responders; DER, distress-endurers; EER, eustress-endurers; FAR, fear-avoiders. *χ*^2^, Chi-Squared; *p, p*-value; *, significant; *V*, Cramer's *V*; no degrees of freedom existent due to Monte Carlo simulation; ^1^*n* = 131 within this category.

In the confirmatory analysis with subgroups based on cut-offs, DERs reported more mental suffering than ARs and EERs. The results regarding the other main categories were not significant in the AEM-subgroups clustering solution nor in the confirmatory analysis.

### Inductive categories: “job stress”, “other workplace problems”, “familial loss”, “coping resources”

Considering the complete sample studied, 32.85% (*n* = 45) were identified to be exposed to “job stress”, and 19.08% (*n *= 25) reported “other workplace problems”. “Familial loss” and accompanying strain was reported by 11.45% (*n* = 15) of the patients, whereas coping resources in daily life were mentioned by 32.3% (*n* = 41) of the patients. For the numbers divided by AEM-subgroups, the results of the Pearson`s chi square tests, and significant differences between subgroups see Table 5. Significant between-group differences were found for “job stress” with DERs indicating that they perceived significantly more “job stress” than ARs (Cramer's *V* = 0.266, indicating a weak effect). Moreover, analysis revealed that FARs reported significantly more “other workplace problems” than ARs, and tendentially to EERs (Cramer's *V* = 0.377, weak to moderate effect). Regarding “familial losses” the DERs reported significantly more “familial losses” compared to ARs (Cramer's *V* = 0.257, indicating a weak effect). On the other hand, the ARs reported significantly more “coping resources” compared to the FARs (Cramer's *V* = 0.345, moderate effect), and ARs tended to have more “coping resources” than DERs. The following coping resources were mentioned: *Exercising/sports* (*n* = 15, e.g., “exercising regularly is good for me”), *hobbies/interests* (*n* = 14, e.g., “taking time to read a book feels good”), *coping with stress/setting boundaries* (*n* = 12, e.g., “I am always able to delegate work”), *positive attitudes* (*n* = 6, e.g., “complaining would only enhance the suffering”), *social environment* (*n* = 6, e.g., “I’ve got a wonderful support system”), *hope/motivation* (*n* = 6, e.g., “it will get better now”), and *external explanatory models* (*n* = 17, e.g., “both my parents also suffered from back pain”).

**Table 5:** Numbers of positive answers of inductive categories per AEM-subgroup and results of Pearson's Chi-Squared tests to compare the subgroups within these categories*.*

	Number of positive answers per subgroup; *n* (%)				Pearson's Chi-Squared tests			Compared cells
Category	FAR	DER	EER	AR	*χ* ^2^	*p*	*V*	
Job stress	9 (27.27)	21 (44.68)	10 (43.48)	5 (14.71)	9.700	**0**.022*	0.266	DER > AR
Other workplace problems	14 (42.42)	6 (12.77)	3 (13.04)	2 (5.88)	17.779	**<0**.001*	0.360	FAR > AR and tendentially EER
Familial loss	5 (15.15)	9 (19.15)	1 (4.35)	0 (0.00)	9.047	**0**.029*	0.257	DER > AR
Coping resources	6 (18.18)	13 (27.66)	3 (13.04)	19 (55.88)	16.335	**0**.001*	0.345	AR > FAR and tendentially DER

AEM, avoidance-endurance model; *n*, number; AR, adaptive responders; DER, distress-endurers; EER, eustress-endurers; FAR, fear-avoiders. *χ*^2^, Chi-Squared; *p, p*-value; *, significant; *V*, Cramer's *V*; no degrees of freedom existent due to Monte Carlo simulation.

In the confirmatory analysis based on cut-offs, the results vary slightly from the cluster solution: regarding “job stress” and “other workplace problems” chi-square tests yielded significant results for the main analysis. However, comparing cells only revealed tendencies for between group differences with FARs tending to perceive more “job stress” than ARs, and DERs tending to suffer more from “other workplace problems” than ARs when compared to the square root of the chi-square critical value of the entire contingency table. Main results considering “coping resources” and “familial loss” revealed no significant differences between subgroups in this cut-offs based analysis.

## Discussion

This study is the first to comprehensively investigate differences in the perception of both psychosocial stressors and availability of coping resources between the AEM-subgroups of CLBP patients. Findings revealed significant differences between subgroups for five of seven categories: “mental suffering”, “job stress”, “other workplace problems”, “familial losses” and “coping resources”, whereas FAR and DER reported higher strain in some life categories (e.g., FAR reported more strain in “mental suffering” and “other workplace problems”, whereas DER reported higher strain in “mental suffering”, “familial loss” and “job stress”). Additionally, the group of ARs with the lowest risk of pain chronification (2, 38) reported a higher quantity of “coping resources”.

Recent evidence conveys that approximately one third of CLBP patients are diagnosed with psychological disorders (9). Among these are anxiety disorders (12.2%), depressive disorders (13.4%), or psychoses (2.4%) (12). The results of our study were consistent with these findings (11, 13); one third of the CLBP patients participating were found to suffer mentally, as they presented at least one main symptom of mental disorders (62). As we hypothesized, the group of FARs and DERs reported significantly more mental suffering than ARs, and FARs significantly more than EERs. These results align with earlier research that found both FARs and DERs experience more affective distress compared to the other subgroups (37). The perceived lower levels of mental suffering experienced by ARs may be furthermore related to the better therapy outcomes experienced by this subgroup (2, 38).

The AEM-groups in this study did not differ in the extent to which they identified job-related strain, even though more than 50% of the total sample study reported job strain, and associations of back pain and work-related stress are consistent with earlier research (16–21). As job strain may be elusive and different aspects may lead to feeling stressed at work, inductive categories were formed from qualitative aspects of job strain. E.g., high work-load, time pressure, high responsibility, and lack of possibilities to relax were named as “job stress” with DERs reporting more job stress than ARs. This might indicate either that ARs experience fewer of these stressors, or that they might be able to cope with such stressors in a better way, possibly because they perceive more available coping resources. Furthermore, patients who are over-active and pain-persistent, tendencies highly comparable to DER (2, 32, 36), were shown to demonstrate higher job stress and to engage in high work-load demands (34, 35). In a second category, dissatisfaction with the workplace, absences and illness, unemployment, avoidance of work, and bad working atmosphere were categorized as “other workplace problems”. Perceived higher levels of other workplace problems amongst FARs is in line with other studies, which found FAR to be associated with work absences and disabilities at work (27, 28), indicating that individuals with higher avoidance-goals likely display higher pain and disability levels (63).

Literature suggests that both family-related and social stressors have an impact on pain chronification, and family was found to be a highly relevant life value in a sample of CLBP patients (64). Approximately one third of all the participants in this study was concerned with family-related or social strain, whereas the respective subgroups were relatively equally affected by family or social strain. In their interviews, these patients identified that they were afflicted with “familial losses” (separation or strain through cases of death), which may in turn show us the importance of social support for an individual's health (65–67). Regarding subgroup differences, the DERs reported significantly more experiences of “familial loss” than ARs. This finding might relate to the higher depression levels observed from in the mental health inventory of DERs.

In addition to identifying differences regarding stressors, this study is also highlights the importance of an individual's perceived coping resources. According to the transactional stress model, the determination of a challenging situation as stressful is based on one's appraisal of their own coping resources (3). Our study found that the ARs identified significantly more external (e.g., exercise/sports, interests and hobbies, the social environment, external explanatory model) and internal resources (e.g., hope/motivation, setting boundaries, and positive attitudes) than FARs, and tendentially more than DERs. Our findings appear to be corroborated by current evidence and suggest that social supports like family and social contacts are helpful with regard to health issues (68–70), and that exercise as well as extracurricular activities (e.g., music-related activities) are an effective way of coping with stress (71–73) and preventing burnout (72).

This study's findings have major implications for goal setting and intervention planning within the different AEM groups of CLBP patients. Cognitive-behavioural interventions have already been evaluated in AEM-subgroups (74). Therefore, it is recommended that FARs receive motivation to be more bodily active, whereas ERs have to learn to set pauses and interrupt tasks if their pain increases. ERs have to train to be aware of their body, and to not overstrain their emotional and bodily limits to complete a task. Likewise, differences in perceived psychosocial stressors and respective compensation strategies using coping resources between AEM- subgroups suggest that subgroup-specific psychological interventions would be appropriate. For instance, interventions should focus on addressing mental suffering and dealing with work problems with FARs and DERs, whereas a resource-centered approach, using internal and external coping resources, would be an appropriate strategy to employ for all subgroups with maladaptive pain coping.

### Limitations

Despite its strengths, this study also has certain limitations. The representativeness of the study sample may be limited due to the low to moderate levels of pain and disability of the participants, although such pain intensity levels on the VAS are typical for chronic pain. Some effects were also quite small so that further research is needed to replicate findings. Another limitation is that to make the study feasible, interviews were not recorded, and therefore only written notes are available, as opposed to audio files and complete transcripts for more in-depth exploration. However, additional inductive categories were built to allow a greater insight and understanding of stressors and coping resources of LBP patients. We used a hierarchical cluster analysis to build the AEM-subgroups, which may be influenced by the individual characteristics of the study population. In order to confirm the validity of the subgroup classification we also used a cut-off-method, which in general revealed comparable results. In some cases, the cut-off method revealed different results for FAR and DER groups than what was found through cluster analysis. This variability may be explained by the possibility that DERs alternate between fear-avoidance and endurance behavior, as has been described in the literature (75, 76). Huijnen (76) described such patients as mixed performers [e.g., in patients demonstrating endurance behaviour, in particular DERs, pain persistence behaviour could cause an increase of pain levels, which may then result in pain-avoidance behaviour and lower drive functions (77)]. However, recent studies strongly support the use of the cluster analysis (52).

## Conclusions

The findings of our study suggest that patients would benefit from subgroup-specific and resource-centered psychological interventions that target maladaptive coping styles. Future research will, however, have to determine more closely if FARs, EERs and DERs as well as ARs also demonstrate their specific behavior patterns in response to other forms of stress or challenging situations, beyond coping with pain.

## Brief summary

This study for the first time comprehensively investigated differences in perceived psychosocial stressors and coping resources between groups of chronic low back pain patients as classified by the Avoidance-Endurance Model. The qualitative as well as quantitative research approach serves to overcome shortcomings associated with a solely quantitative approach and thus gives a more detailed insight into relevant psychosocial stressors and coping resources in patients with different pain behaviour patterns. As results show significant differences between groups, they are of high relevance in the context of preventing pain chronification and highlight the importance of individual coping behaviours as well as coping resources. This study thus sheds light on psychosocial dimensions of pain and provides helpful information for health professionals of variable professions regarding individualized therapy planning especially for groups of patients with maladaptive pain coping behaviours. It also stresses the importance of coping resources as protective factors against pain chronification as well as possible starting points for resource-centred therapy.

## Data Availability

The raw data supporting the conclusions of this article will be made available by the authors, without undue reservation.
